# The impact of nursing intervention based on teach-back method on self-management behavior and negative emotions of postoperative patients with aortic dissection

**DOI:** 10.3389/fcvm.2025.1631750

**Published:** 2026-06-24

**Authors:** Qin Zeng, Xiangrong Li, Yang Liu

**Affiliations:** 1 Chest Surgery, Wuhan Jinyintan Hospital, Wuhan, China; 2 Trauma Surgery, Wuhan Jinyintan Hospital, Wuhan, China

**Keywords:** nursing intervention, teach-back method, self-management behavior, negative emotions, aortic dissection

## Abstract

**Background:**

Aortic dissection (AD) was a severe cardiovascular condition requiring comprehensive postoperative management. The teach-back method, a patient-centered educational strategy, may enhance patient comprehension and adherence to self-management practices post-surgery, potentially reducing negative emotions and improving outcomes. This study aimed to evaluate the impact of the teach-back method on self-management behaviors and negative emotions in postoperative AD patients at a tertiary hospital in China.

**Methods:**

This retrospective cohort study was conducted at The First Affiliated Hospital of Nanchang University, China. It analyzed 210 postoperative AD patients from March 2021 to March 2023, divided evenly into a routine nursing group and a teach-back method group. Various assessments, including the Health Education Knowledge Acquisition Survey, Visual Analog Scale (VAS), Hamilton Anxiety (HAMA) and Depression (HAMD) Scales, Self Care Level Scale (ESCA), and WHO Quality of Life Inventory (WHOQOL-BREF), were performed pre- and post-intervention. Between-group comparisons were performed using independent *t*-tests or chi-square tests, with a significance level of *P* < 0.05.

**Results:**

Patients in the teach-back group showed higher mastery of health education knowledge (96.19% vs. 88.57%; *χ*^2^ = 4.33, *P* = 0.037) and significant reduction in anxiety levels [HAMA: 11.85 ± 4.00 [11.08, 12.62] vs. 13.15 ± 4.92 [12.20, 14.10]; *t* = 2.097, *P* = 0.037]. Self-management scores improved significantly in knowledge level and self-protective skills, with mean scores higher in the teach-back group (*P* < 0.05). However, no significant differences were observed in pain scores or postoperative complications between the groups.

**Conclusion:**

The teach-back method significantly enhances knowledge acquisition and anxiety reduction in postoperative AD patients, showing potential as a beneficial adjunct to routine nursing care.

## Introduction

Aortic dissection (AD) represents a life-threatening cardiovascular condition characterized by the separation of the aortic wall layers, leading to a false lumen formation (1). The incidence of AD was approximately 2.9 cases per 100,000 person-years, and it remains a significant cause of morbidity and mortality despite advancements in surgical and medical management (2). The postoperative management of patients with AD was complex, requiring meticulous attention to blood pressure control, pain management, and prevention of complications, which mandates a robust understanding and adherence to self-management practices (3).

Effective patient education was a cornerstone of postoperative care in AD, promoting improved clinical outcomes and quality of life by empowering patients to actively participate in their recovery process (4). Traditional patient education approaches, often unidirectional and rote, have shown limitations in ensuring adequate comprehension and retention of health information, impeding the adoption of effective self-care behaviors (5, 6). This gap in patient understanding has been associated with an increased risk of postoperative complications, hospital read missions, and a deterioration in patient well-being (7). Thus, exploring innovative educational interventions that prioritize patient engagement and understanding was paramount to optimizing postoperative care in this cohort (8).

The teach-back method emerges as a promising patient-centered educational strategy designed to bridge the gap between healthcare provider instruction and patient comprehension (9). This iterative process involves patients repeating the information provided to them in their own words, enabling healthcare practitioners to confirm understanding and correct any misconceptions timely (10, 11). Empirical evidence suggests that the teach-back method enhances patient knowledge retention and health literacy across various patient populations, including those suffering from chronic conditions (12). For instance, studies in diabetes and heart failure populations have demonstrated improved self-care and reduced readmission rates with teach-back interventions. Despite the growing body of literature advocating for the teach-back method, its application and efficacy in postoperative AD patients remain inadequately explored (13).

Given these considerations, this study seeks to evaluate the impact of a nursing intervention based on the teach-back method on self-management behaviors and negative emotions in postoperative AD patients.

## 2 Materials and methods

### 2.1 Study design and participant allocation

This retrospective cohort study enrolled 210 consecutive patients diagnosed with aortic dissection (AD) who underwent surgical intervention at our institution between March 2021 and March 2023. During this period, a structured teach-back method was progressively integrated into the standard nursing protocol. From March 2021 to February 2022, all eligible patients received routine nursing care (Routine Group, *n* = 105). From March 2022 to March 2023, all subsequent eligible patients received the teach-back method in addition to routine care (Teach-back Group, *n* = 105). This sequential allocation was based on the implementation timeline of the new educational strategy and was not randomized. All patients met the same inclusion and exclusion criteria throughout the study period. A total of 245 patients were assessed for eligibility. Thirty-five patients were excluded: 12 did not meet inclusion criteria, 15 had incomplete data, and 8 declined participation. The remaining 210 patients were allocated sequentially into the two groups as described above, ensuring no overlap in the intervention periods.

Demographic data, such as general information and postoperative complications, were collected via the medical record system. The study analyzed the patients' comprehension of health knowledge, pain scores, and quality of life, as well as their self-management behaviors and negative emotions.

The study received approval from the Institutional Review Board and Ethics Committee of Wuhan jinyintan hospital. Due to its retrospective nature, informed consent was waived, as only de-identified patient data were used, ensuring no risk or impact on patient care. This waiver aligns with regulatory and ethical guidelines for retrospective research and was sanctioned by the Institutional Review Board and Ethics Committee. All assessment tools used in this study are standardized and publicly available instruments. Permission for their use was not required as they are widely used in clinical research and have been validated in Chinese populations.

### 2.2 Inclusion and exclusion criteria

**Inclusion criteria:** 1) Patients who meet the clinical diagnostic criteria for AD; 2) Availability of complete clinical data; 3) Normal cognitive and communication abilities; 4) Good compliance with treatment; 5) No known allergies to anesthetic drugs.

**Exclusion criteria:** 1) Patients with other serious illnesses that prevent cooperation with the study; 2) Evidence of mental disorders; 3) Lack of a clear treatment plan.

### 2.3 Treatment approach

#### 2.3.1 Routine nursing group

The routine nursing group received standard health education, focusing on six main aspects: assessment phase, psychosocial support, disease-specific nursing, symptom management, daily management, and continuity of care.

Assessment Phase: Face-to-face interviews were conducted to inform patients about the interview process and objectives. Baseline information such as occupation, education level, and personality traits were collected. This information was used to evaluate the patients' cognitive and acceptance abilities, forming the basis for developing individualized health education plans. Psychosocial Support: Patients and their families were assessed for negative emotions like anxiety, worry, and helplessness. An understanding of the family's economic situation and social support systems was gained. The importance of family support was emphasized, advising families to provide companionship and encouragement to boost patients' confidence in their treatment. Disease-specific Nursing: An educational manual on AD was provided, covering its pathogenesis, treatment options, and rehabilitation management. Based on the cognitive assessment, the assigned nurse delivered tailored explanations to enhance patients' understanding of the disease, thereby improving cooperation during treatment and nursing. Symptom Management: Pain Management. A thorough assessment of pain location, nature, duration, and triggers was conducted. Patients were guided to avoid exertion and use analgesics as prescribed, with monitoring of pain relief and side effects to optimize the analgesic regimen. Blood Pressure Stabilization. Blood pressure changes were closely monitored, and triggers like stress and pain were addressed. Strict control of fluid intake and administration was maintained, with sedatives and analgesics given as prescribed for stress-induced hypertension. Daily Management. A quiet and comfortable hospital environment was maintained, with patients instructed to have complete bed rest and ensure adequate sleep. Families were informed about collaborating effectively while avoiding emotional fluctuations in patients. Patients were advised to consume a high-protein, high-fiber diet that was light and easy to digest to prevent constipation. Continuity of Care: Establishment of Health Management Records. During hospitalization, relevant patient data were collected to create a health management record, which included basic information, treatment history, disease awareness, self-care level, and follow-up appointment schedules. Discharge Health Education. On discharge day, an AD continuing health education manual was provided, featuring guidelines on healthy lifestyles, self-monitoring of blood pressure, emergency measures, and follow-up instructions. Patients were also instructed to keep a daily log of their health status. Post-discharge Follow-up. Monthly telephone follow-ups were conducted over three months to comprehensively assess patients' living situations and self-management after discharge.

#### 2.3.2 Experimental group

In addition to the standard methods, this group employed a health education approach based on the teach-back method. The details were as follows (14, 15).

**Establishment of a Dedicated Teach-Back Health Education Team:** To address the specific needs of post-AD surgery patients, a specialized team comprising six experienced nurses was formed. These nurses underwent targeted skills training. The team was tasked with designing and implementing the teach-back health education plan and evaluating its outcomes. Through discussions and interviews, the team sought to gain a comprehensive understanding of the patients' knowledge, needs, and concerns regarding AD, enabling the development of a tailored health education program. The team members received training from a specialist physician, who provided insights into the surgery's objectives, indications, preoperative preparation, surgical procedures, and postoperative care. In addition, the physician addressed common patient queries and shared clinical experiences and effective health education strategies. **Development of Educational Brochures:** Brochures were created to provide patients with guidance and advice, covering the mechanisms of the disease, treatment options, and rehabilitation management. These were distributed to the patients to enhance their understanding and engagement. **Specific Approach:** Effective Explanation. Nurses explained AD -related knowledge to patients using simple and comprehensible language. Proper Assessment. Patients were given 1–2 days to process the information independently. One-on-one interviews were conducted where patients were encouraged to describe the content in their own words, helping evaluate their grasp of physiological knowledge and preparing for subsequent health education. Clarification and Correction. For any errors or incomplete understanding in patients' responses, nurses provided additional explanations using visual aids, multimedia, or various methods to facilitate better comprehension. Accurate Understanding. Patients were asked to reiterate the explained content, and any misunderstandings were further clarified and corrected until accurate understanding was achieved. Continuity of Care. Monthly telephone follow-ups, conducted over three months, were used to assess patients' post-discharge lives and self-management. Any issues encountered at home were promptly addressed, and reminders were given to adhere to prescribed medications and attend timely follow-up consultations.

### 2.4 Health education knowledge acquisition

Eight weeks post-surgery, patients completed the Health Education Knowledge Acquisition Survey, a locally developed instrument comprising 21 items covering specialized disease knowledge, pain management, daily management, and discharge health education. Each item is scored on a 3-point scale (0 = not understood, 1 = partially understood, 2 = fully understood), with total scores categorized as “fully acquired” (≥38), “partially acquired” (20–37), and “not acquired” (<20). The instrument demonstrated good internal consistency in our sample (Cronbach's *α* = 0.82). The rate of knowledge acquisition was calculated using the formula: Knowledge Acquisition Rate = (Fully Acquired + Partially Acquired)/Total Number of Cases × 100%.

### 2.5 Pain assessment

The Visual Analogue Scale (VAS) was used to evaluate the intensity of pain experienced by patients both before and after receiving nursing care, with assessments conducted one week and eight weeks post-surgery, respectively. The VAS measures pain on a 10-centimeter scale, with higher scores indicating more intense pain. Patients assessed their pain levels at rest, during daily activities, and at night. They marked their pain intensity on a line ranging from “0”, indicating “no pain”, to “10”, indicating “the worst pain imaginable”. The distance from “0” to the point marked by the patients was measured to determine their pain score. This scale was particularly suitable for adult patients, with a Cronbach's alpha coefficient of 0.86, demonstrating reliable internal consistency (16).

### 2.6 Psychological state assessment

The Hamilton Anxiety Scale (HAMA) and the Hamilton Depression Scale (HAMD), Chinese versions validated in cardiac populations, were employed to assess patients' anxiety and depression levels one week and eight weeks post-surgery, respectively. HAMA consists of 14 items, each rated from 0 to 4 according to specific guidelines that denote the severity of symptoms: 0 for no symptoms, 1 for mild symptoms occurring irregularly, 2 for moderate symptoms that occur more frequently and require effort to manage, 3 for severe symptoms that were continuous and dominating, and 4 for very severe, incapacitating symptoms. These items cover groups of symptoms such as autonomic and respiratory symptoms, as well as fears, rather than individual symptoms. A HAMA score of 21 or higher, and a HAMD score of 18 or higher, indicates significant anxiety or depression, respectively. The total score ranges from 0 to 56, with higher scores reflecting more severe negative emotions. The scale's internal consistency was robust, with a Cronbach's alpha coefficient of 0.905 (17).

### 2.7 Self-management level assessment

The Self Care Level Scale (ESCA) was utilized as previously validated in Chinese patients with chronic diseases to assess the self-management levels of patients one week and eight weeks post-surgery, respectively. The ESCA comprises four dimensions: self-concept (9 items, totaling 36 points), self-responsibility (8 items, totaling 32 points), self-care skills (12 items, totaling 48 points), and health knowledge level (14 items, totaling 56 points), amounting to a total of 43 items. The scoring follows a three-level method, with a total possible score ranging from 0 to 172. Higher scores indicate stronger self-care abilities. The scoring classification was as follows: low (0–57 points), medium (58–115 points), and high (116–172 points). The scale demonstrated good internal consistency, with a Cronbach's alpha coefficient of 0.78 (18).

### 2.8 Quality of life assessment

The World Health Organization Quality of Life Inventory (WHOQOL-BREF), Chinese version, was used to assess quality of life. It contains 26 items covering four domains: physical health (7 items), psychological health (6 items), social relationships (3 items), and environment (8 items). Each item is rated on a 5-point Likert scale. Domain scores are transformed to a 0–100 scale, with higher scores indicating better quality of life. The instrument has been validated in Chinese cardiac populations and showed excellent internal consistency (Cronbach's *α* = 0.9685) in this study (19). We consistently refer to the two assessment time points as “Baseline (1 week post-surgery)” and “Follow-up (8 weeks post-surgery)”. All measures (VAS, HAMA, HAMD, ESCA, WHOQOL-BREF) were collected at these two time points. There was no attrition or missing data for the primary outcomes, as all 210 participants completed both assessments. In the ESCA scale, the four dimensions are labeled consistently as: Responsibility, Knowledge Level, Self-Care Skills, and Self-Care Concepts (formerly referred to as “self-protective skills” and “self-protection concept” in the original submission).

### 2.9 Statistical analysis

Data were analyzed using SPSS 29.0 and R software (version 4.3.1). Continuous variables were tested for normality with the Shapiro–Wilk test. Between-group comparisons of baseline characteristics used independent *t*-tests or Mann–Whitney *U*-tests for continuous variables, and chi-square or Fisher's exact tests for categorical variables.

For longitudinal outcomes (VAS, HAMA, HAMD, ESCA, WHOQOL-BREF), we employed repeated-measures analysis of variance (RM-ANOVA) to examine the effects of time, group, and time-by-group interaction. When sphericity was violated, Greenhouse–Geisser corrections were applied. *Post-hoc* analyses with Bonferroni adjustment were conducted for significant interactions. Effect sizes are reported as partial eta-squared (*η*^2^) for ANOVA models and Cohen's *d* for pairwise comparisons, with 95% confidence intervals (CIs). A two-tailed *p*-value < 0.05 was considered statistically significant. Given the multiple outcome comparisons, we also applied the False Discovery Rate (FDR) correction using the Benjamini–Hochberg procedure. Correlation analysis was performed using Pearson correlation for continuous variables and Spearman correlation for categorical variables.

## 3 Results

### 3.1 Baseline and clinical characteristics

The baseline demographic and clinical characteristics of the 210 participants are summarized in Table 1. Both groups were well-matched in terms of age, gender, education, and comorbidities (all *p* > 0.05). The majority of patients had Stanford type A dissection (Routine: 54.3%; Teach-back: 59.0%, *p* = 0.486) and underwent similar surgical procedures (e.g., Bentall, TEVAR). However, detailed intraoperative data such as cardiopulmonary bypass time, specific surgical techniques, and postoperative analgesia protocols were not systematically recorded in the electronic medical records for this retrospective cohort. Similarly, ICU and hospital length of stay data were incomplete and thus not included in the analysis. Future prospective studies will aim to collect these important clinical parameters to better control for potential confounders.

**Table 1:** Baseline and clinical characteristics.

Parameters	Routine nursing (*n* = 105)	Teach-back method (*n* = 105)	*t*/*χ*^2^	*P*
Gender (Male/Female)	80 (76.19%)/25 (23.81%)	78 (74.29%)/27 (25.71%)	0.102	0.749
Age (years)	56.34 ± 12.75	57.26 ± 12.44	0.531	0.596
Degree of education (Junior high school and below/technical secondary school/high school/junior college/undergraduate)	82 (78.1%)/2 (1.9%)/8 (7.62%)/5 (4.76%)/8 (7.62%)	85 (80.95%)/3 (2.86%)/10 (9.52%)/2 (1.9%)/5 (4.76%)	2.454	0.653
Body Mass Index (kg/m^2^)	25.00 ± 3.99	26.28 ± 14.78	0.861	0.391
Stanford type (A/B) (A/B)	57 (54.29%)/48 (45.71%)	62 (59.05%)/43 (40.95%)	0.485	0.486
Smoking history [*n* (%)] (Yes/No)	50 (47.62%)/55 (52.38%)	57 (54.29%)/48 (45.71%)	0.934	0.334
Marital Status (Married/Single/Divorce/Widowed or widowed/Others)	94 (89.52%)/8 (7.62%)/2 (1.9%)/0 (0%)/1 (0.95%)	97 (92.38%)/4 (3.81%)/2 (1.9%)/1 (0.95%)/1 (0.95%)	3.156	0.723
Primary caregiver (parent/spouse/children/others)	10 (9.52%)/61 (58.1%)/28 (26.67%)/6 (5.71%)	5 (4.76%)/53 (50.48%)/40 (38.1%)/7 (6.67%)	4.423	0.219
Drinking history [*n* (%)] (Yes/No)	61 (58.1%)/44 (41.9%)	57 (54.29%)/48 (45.71%)	0.310	0.578
Employment [*n* (%)] (Yes/No)	46/59	48/57	0.077	0.781
Diabetes (Yes/No)	27/78	26/79	0.025	0.874
Hypertension (Yes/No)	80 (76.19%)/25 (23.81%)	83 (79.05%)/22 (20.95%)	0.247	0.619
Residence Area (town/rural)	49 (46.67%)/56 (53.33%)	53 (50.48%)/52 (49.52%)	0.305	0.581
live alone (Yes/No)	31 (29.52%)/74 (70.48%)	29 (27.62%)/76 (72.38%)	0.093	0.760

Data are presented as mean ± standard deviation or *n* (%). Between-group comparisons used independent *t*-tests for continuous variables and chi-square or Fisher's exact tests for categorical variables.

### 3.2 Mastery of health education knowledge

The teach-back method group exhibited a higher rate of full mastery, with 58 patients (55.24%) fully mastering the material, compared to 42 patients (40.00%) in the routine nursing group (Table 2). The mastery rate, defined as the percentage of patients achieving either basic or full mastery, was 96.19% in the teach-back method group, compared to 88.57% in the routine nursing group, reflecting a statistically significant difference (*χ*^2^ = 4.33, *P* = 0.037). This indicates that the teach-back method was more effective in enhancing patients' understanding of health education knowledge postoperatively.

**Table 2:** Comparison of mastery of health education knowledge between two groups of patients.

Parameters	Routine nursing (*n* = 105)	Teach-back method (*n* = 105)	χ^2^	*P*
Not mastered	12	4		
Basic mastery	51	43		
Fully mastered	42	58		
Mastery rate [*n* (%)]	93 (88.57%)/12 (11.43%)	101 (96.19%)/4 (3.81%)	4.33	0.037

Mastery rate = (Fully Acquired + Partially Acquired)/Total Number of Cases × 100%. Between-group comparison used chi-square test.

### 3.3 Pain scores

Pre-intervention VAS scores were similar, with means of 7.44 ± 1.63 [7.13, 7.75] in the routine nursing group and 7.45 ± 1.11 [7.24, 7.66] in the teach-back method group (*t* = 0.076, *P* = 0.939) (Table 3). Post-intervention VAS scores were also comparable, with means of 2.36 ± 0.23 [2.32, 2.40] for the routine nursing group and 2.32 ± 0.16 [2.29, 2.35] for the teach-back method group (*t* = 1.608, *P* = 0.109). These results suggest that both nursing interventions were equally effective in reducing postoperative pain.

**Table 3:** Comparison of VAS pain scores before and after nursing interventions in two patient groups.

Parameters	Routine nursing (*n* = 105)	Teach-back method (*n* = 105)	*t*	*P*
Pre-VAS	7.44 ± 1.63 [7.13, 7.75]	7.45 ± 1.11 [7.24, 7.66]	0.076	0.939
Post-VAS	2.36 ± 0.23 [2.32, 2.40]	2.32 ± 0.16 [2.29, 2.35]	1.608	0.109

VAS, Visual Analog Scale. Data are presented as mean ± standard deviation [95% confidence interval]. Between-group comparisons used independent *t*-tests.

### 3.4 Postoperative complications

In the routine nursing group, complications included wound infection (*n* = 2), pulmonary infection (*n* = 7), and pressure injury (*n* = 1), resulting in an overall incidence rate of 9.52% (Table 4). In comparison, the teach-back method group reported 1 wound infection, 6 pulmonary infections, and 1 pressure injury, with an overall incidence rate of 7.62%. Statistical analysis revealed no significant difference between the groups in terms of complication rates (*χ*^2^ = 0.243, *P* = 0.622), indicating similar effectiveness of both nursing interventions in mitigating postoperative complications following AD surgery.

**Table 4:** Comparison of postoperative complications between two patient groups.

Parameters	Routine nursing (*n* = 105)	Teach-back method (*n* = 105)	χ^2^	*P*
Wound infection	2	1		
Pulmonary infection	7	6		
Pressure injury	1	1		
Incidence rate [*n* (%)]	10 (9.52%)/95 (90.48%)	8 (7.62%)/97 (92.38%)	0.243	0.622

Between-group comparison used chi-square test.

### 3.5 Hama and Hamd Scores

**Figure 1:**
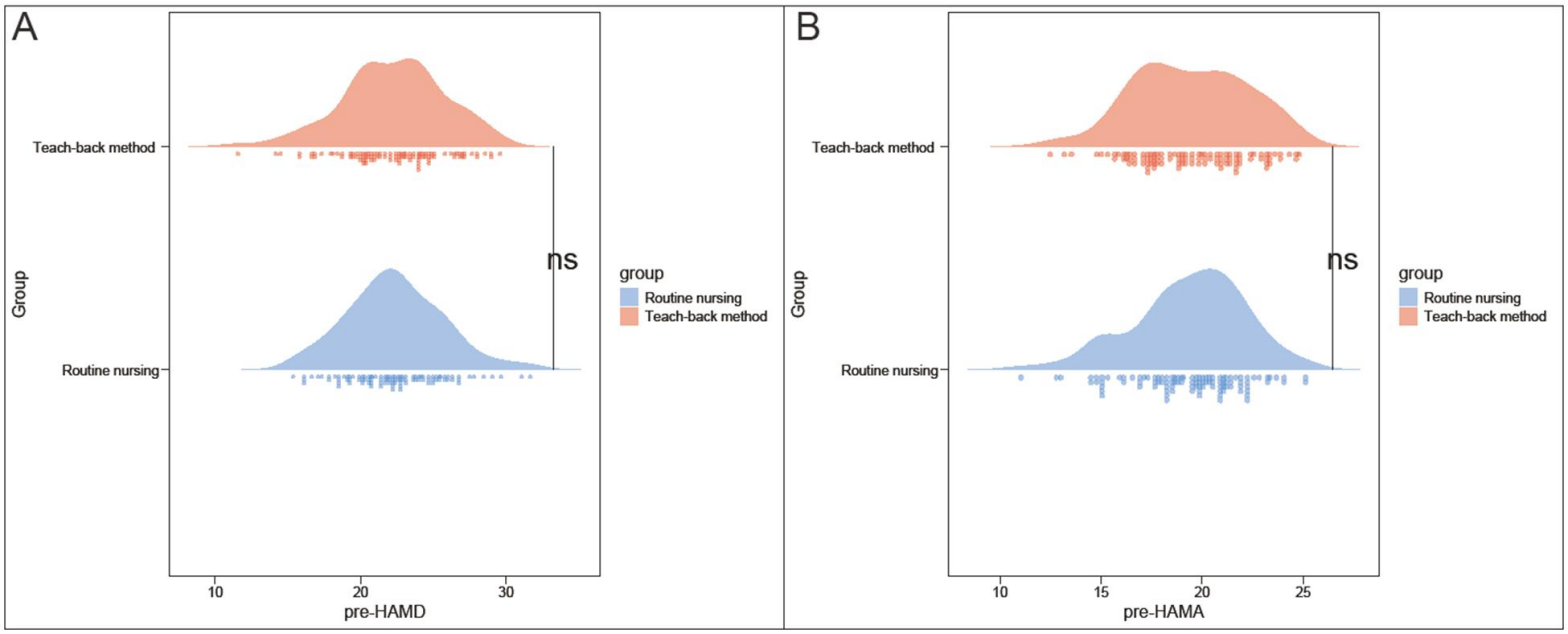
Comparison of pre-nursing HAMA and HAMD scores between two patient groups. **(A)** HAMD scores; **(B)** HAMA scores.

The mean HAMA scores were 19.36 ± 2.75 [18.83, 19.89] for the routine nursing group and 19.44 ± 2.77 [18.91, 19.97] for the teach-back method group (*t* = 0.208, *P* = 0.836), while the mean HAMD scores were 22.37 ± 3.26 [21.74, 23.00] and 22.30 ± 3.49 [21.62, 22.98], respectively (*t* = 0.160, *P* = 0.873) (Figure 1). These results suggest that baseline levels of anxiety and depression were comparable between the two groups prior to the implementation of the nursing interventions.

**Figure 2:**
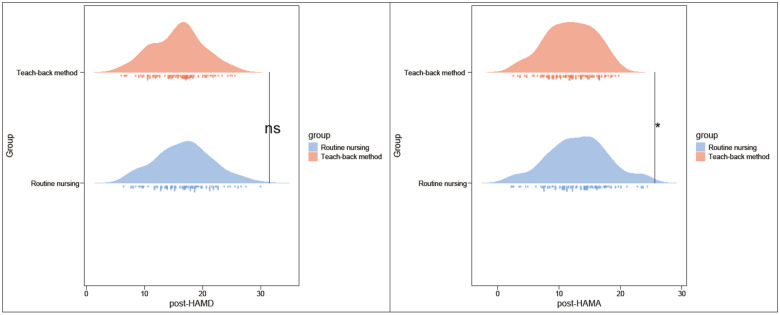
Comparison of post-nursing HAMA and HAMD scores between two patient groups. **(A)** HAMD scores; **(B)** HAMA scores.

Following the nursing interventions, the teach-back method group demonstrated a statistically significant reduction in anxiety levels compared to the routine nursing group, as measured by the HAMA Rating Scale (Figure 2). The mean post-nursing HAMA scores were 13.15 ± 4.92 [12.20, 14.10] in the routine nursing group and 11.85 ± 4.00 [11.08, 12.62] in the teach-back method group, with this difference reaching statistical significance (*t* = 2.097, *P* = 0.037). Conversely, while there was a reduction in depression levels as assessed by the Hamilton Depression Rating Scale (HAMD), the difference between the two groups was not statistically significant. The mean HAMD scores post-nursing were 16.81 ± 4.87 [15.87, 17.75] for the routine nursing group and 15.76 ± 4.36 [14.92, 16.60] for the teach-back method group (*t* = 1.656, *P* = 0.099). This suggests that while the teach-back method had a significant impact on reducing anxiety, its effect on depression, although present, did not reach statistical significance.

### 3.6 ESCA

The mean scores for responsibility were 18.21 ± 2.25 [17.78, 18.64] in the routine nursing group and 18.19 ± 2.05 [17.79, 18.59] in the teach-back method group (*t* = 0.055, *P* = 0.956) (Table 5). Similarly, the knowledge level scores were 31.57 ± 3.06 [30.98, 32.16] and 31.76 ± 3.93 [31.00, 32.52], respectively (*t* = 0.401, *P* = 0.689). Scores for self-protective skills (25.36 ± 3.42 [24.70, 26.02] vs. 25.16 ± 3.21 [24.54, 25.78]; *t* = 0.442, *P* = 0.659) and self-protection concept (19.95 ± 3.67 [19.24, 20.66] vs. 19.86 ± 3.26 [19.23, 20.49]; *t* = 0.180, *P* = 0.857) also demonstrated no significant differences between the two groups. These results indicate that both groups had similar baseline levels of self-management skills prior to the nursing interventions.

**Table 5:** Comparison of pre-nursing ESCA dimension scores between two patient groups.

Parameters	Routine nursing (*n* = 105)	Teach-back method (*n* = 105)	*t*	*P*
Responsibility	18.21 ± 2.25 [17.78, 18.64]	18.19 ± 2.05 [17.79, 18.59]	0.055	0.956
Knowledge level	31.57 ± 3.06 [30.98, 32.16]	31.76 ± 3.93 [31.00, 32.52]	0.401	0.689
Self protective skills	25.36 ± 3.42 [24.70, 26.02]	25.16 ± 3.21 [24.54, 25.78]	0.442	0.659
Self protection concept	19.95 ± 3.67 [19.24, 20.66]	19.86 ± 3.26 [19.23, 20.49]	0.180	0.857

ESCA, Self Care Level Scale. Data are presented as mean ± standard deviation [95% confidence interval]. Between-group comparisons used independent *t*-tests.

The teach-back method group showed superior performance in knowledge level, with a mean score of 43.98 ± 5.21 [42.97, 44.99] compared to 42.36 ± 4.21 [41.54, 43.18] in the routine nursing group (*t* = 2.484, *P* = 0.014) (Table 6). Additionally, self-protective skills were significantly higher in the teach-back method group, with mean scores of 31.76 ± 4.28 [30.93, 32.59] vs. 30.15 ± 3.50 [29.47, 30.83] for the routine nursing group (*t* = 2.980, *P* = 0.003). The self-protection concept was also enhanced in the teach-back method group, achieving a mean score of 22.37 ± 3.61 [21.67, 23.07] compared to 21.19 ± 2.99 [20.61, 21.77] in the routine group (*t* = 2.571, *P* = 0.011). However, the difference in responsibility scores was not statistically significant, with mean scores of 25.33 ± 3.02 [24.75, 25.91] for the teach-back group and 25.06 ± 2.56 [24.57, 25.55] for the routine group (*t* = 0.702, *P* = 0.484). These findings suggest the teach-back method was more effective in improving certain aspects of self-care management postoperatively.

**Table 6:** Comparison of post-nursing ESCA dimension scores between two patient groups.

Parameters	Routine nursing (*n* = 105)	Teach-back method (*n* = 105)	*t*	*P*
Responsibility	25.06 ± 2.56 [24.57, 25.55]	25.33 ± 3.02 [24.75, 25.91]	0.702	0.484
Knowledge level	42.36 ± 4.21 [41.54, 43.18]	43.98 ± 5.21 [42.97, 44.99]	2.484	0.014
Self protective skills	30.15 ± 3.50 [29.47, 30.83]	31.76 ± 4.28 [30.93, 32.59]	2.980	0.003
Self protection concept	21.19 ± 2.99 [20.61, 21.77]	22.37 ± 3.61 [21.67, 23.07]	2.571	0.011

ESCA, Self Care Level Scale. Data are presented as mean ± standard deviation [95% confidence interval]. Between-group comparisons used independent *t*-tests.

### 3.7 WHOQOL-BREF

The psychological dimension had mean scores of 11.06 ± 2.97 [10.48, 11.64] for the routine nursing group and 11.22 ± 3.01 [10.64, 11.80] for the teach-back group (*t* = 0.381, *P* = 0.703) (Table 7). Physiology scores were 10.26 ± 2.65 [9.75, 10.77] and 10.36 ± 2.45 [9.89, 10.83], respectively (*t* = 0.285, *P* = 0.776). Similarly, social relations scores showed no significant difference, with 10.33 ± 3.01 [9.75, 10.91] in the routine group vs. 10.32 ± 2.81 [9.78, 10.86] in the teach-back group (*t* = 0.015, *P* = 0.988). The environment dimension scores were also comparable, with means of 11.96 ± 2.53 [11.46, 12.46] for the routine group and 11.93 ± 2.56 [11.43, 12.43] for the teach-back group (*t* = 0.065, *P* = 0.948). These results indicate that both groups had similar quality of life perceptions before receiving the respective nursing interventions.

**Table 7:** Comparison of pre-nursing WHOQOL-BREF dimension scores between two patient groups.

Parameters	Routine nursing (*n* = 105)	Teach-back method (*n* = 105)	*t*	*P*
Psychology	11.06 ± 2.97 [10.48, 11.64]	11.22 ± 3.01 [10.64, 11.80]	0.381	0.703
Physiology	10.26 ± 2.65 [9.75, 10.77]	10.36 ± 2.45 [9.89, 10.83]	0.285	0.776
Social relations	10.33 ± 3.01 [9.75, 10.91]	10.32 ± 2.81 [9.78, 10.86]	0.015	0.988
Environment	11.96 ± 2.53 [11.46, 12.46]	11.93 ± 2.56 [11.43, 12.43]	0.065	0.948

Data are presented as mean ± standard deviation (95% confidence interval). Independent samples *t*-test was used for between-group comparisons.

The psychology scores averaged 15.96 ± 2.78 [15.43, 16.49] for the routine nursing group and 16.33 ± 3.46 [15.66, 17.00] for the teach-back method group (*t* = 0.847, *P* = 0.398) (Table 8). In the physiology dimension, scores were 17.06 ± 2.71 [16.54, 17.58] for the routine group and 17.25 ± 3.62 [16.55, 17.95] for the teach-back group (*t* = 0.422, *P* = 0.674). Social relations showed mean scores of 17.67 ± 3.15 [17.06, 18.28] for the routine nursing group and 17.35 ± 2.63 [16.84, 17.86] for the teach-back group (*t* = 0.778, *P* = 0.438). The environment dimension scores, although not reaching statistical significance, showed a trend with mean scores of 17.52 ± 4.07 [16.73, 18.31] for the routine group and 18.56 ± 3.75 [17.83, 19.29] for the teach-back group (*t* = 1.925, *P* = 0.056). These findings suggest that while the teach-back method did not significantly enhance perceived quality of life over routine nursing in most dimensions, it may have a potential effect on environmental quality perceptions.

**Table 8:** Comparison of post-nursing WHOQOL-BREF dimension scores between two patient groups.

Parameters	Routine nursing (*n* = 105)	Teach-back method (*n* = 105)	*t*	*P*
Psychology	15.96 ± 2.78 [15.43, 16.49]	16.33 ± 3.46 [15.66, 17.00]	0.847	0.398
Physiology	17.06 ± 2.71 [16.54, 17.58]	17.25 ± 3.62 [16.55, 17.95]	0.422	0.674
Social relations	17.67 ± 3.15 [17.06, 18.28]	17.35 ± 2.63 [16.84, 17.86]	0.778	0.438
Environment	17.52 ± 4.07 [16.73, 18.31]	18.56 ± 3.75 [17.83, 19.29]	1.925	0.056

Data are presented as mean ± standard deviation (95% confidence interval). Independent samples *t*-test was used for between-group comparisons.

## 4 Discussion

Our findings indicate that the teach-back method was associated with improved knowledge acquisition and reduced anxiety levels compared to routine nursing alone, consistent with the primary outcomes of this study. The results demonstrate the efficacy of a nursing intervention utilizing the teach-back method in enhancing self-management behaviors and attenuating negative emotions among postoperative patients with AD, directly aligning with the statistical findings presented. One of the most notable outcomes from the study was the improved mastery of health education knowledge among patients in the teach-back method group. The reason behind this improvement can be attributed to the interactive nature of the teach-back method, wherein patients were encouraged to articulate their understanding of the health information in their own words (20). This not only reinforces learning but also allows healthcare providers to identify and address any misconceptions immediately (21). By engaging patients actively, the teach-back method facilitates more effective cognitive processing and retention of information (22). This aligns with established learning theories which suggest that active involvement in the learning process enhances comprehension and recall (23). Consequently, this method can be an invaluable tool in managing chronic conditions like AD, which requires patients to have a solid understanding of complex self-care regimens.

However, it is crucial to interpret these findings with caution. Given the non-randomized, retrospective design of this study, the observed associations should not be interpreted as causal. The sequential allocation of participants based on the implementation timeline introduces a significant risk of confounding. For instance, temporal trends in overall care quality, changes in hospital protocols, or a Hawthorne effect—whereby patients in the later (teach-back) group may have altered their behavior simply because they were aware of being part of a new initiative—could partially account for the differences observed. Furthermore, the potential for selection bias and unmeasured confounders (e.g., subtle differences in nursing staff enthusiasm or experience between the two periods) cannot be ruled out. The fragility of some statistically significant results, particularly those with p-values close to 0.05 (e.g., HAMA, *P* = 0.037) and modest effect sizes, underscores the need for a conservative interpretation. These findings represent promising associations that warrant further validation in more rigorous studies.

Furthermore, the teach-back method led to a significant reduction in anxiety levels, as evidenced by the lower HAMA Scale scores post-intervention. This reduction can be partly attributed to the enhanced communication and personalized support provided by the specialized teach-back health education team. By ensuring patients have a clear and accurate understanding of their condition, treatment options, and self-care practices, we can alleviate the uncertainty and apprehension that often accompanies their postoperative experience. Moreover, the repetitive nature of the teach-back method can enhance patient confidence in their knowledge and self-care abilities, thereby reducing feelings of helplessness and anxiety (24, 25). This mirrors the cognitive-behavioral approach, where correcting cognitive distortions and enhancing self-efficacy can significantly alleviate anxiety and other psychological distress.

The modest effect sizes and the lack of a statistically significant effect on depression scores (HAMD, *P* = 0.099) highlight the need for caution in overinterpreting the findings and suggest that the impact may be specific to anxiety or limited in magnitude.In terms of self-management, the teach-back method notably improved aspects such as self-protective skills and self-protection concepts. The underlying mechanism here likely relates to the method's capability to cultivate a deeper, personalized understanding of health information, prompting patients to make more informed decisions regarding their daily health practices (26, 27). This was crucial in managing a condition like AD, where precise adherence to medication, lifestyle modifications, and monitoring for potential complications were imperative (28). The ability to correct misunderstandings through the teach-back process ensures that patients were not merely following instructions, but were aware of the rationale behind them, thus fostering intrinsic motivation and commitment to their health management.

Future randomized controlled trials are warranted to confirm these preliminary observations, control for potential confounders, and explore the mechanisms underlying the observed associations. Interestingly, the study also revealed an improvement in knowledge levels among the teach-back group when measured by the Self-Care Scale (ESCA). Increased knowledge scores indicate that the teach-back method not only assists in the practical application of knowledge but also enhances the patients’ comprehension of the complex interplay between their condition and required lifestyle changes. This deeper understanding can serve as a strong foundation for long-term self-management, reducing the likelihood of rehospitalization and other adverse health outcomes associated with poor self-management practices.

Although depression levels improved slightly, the difference did not reach statistical significance, suggesting that while the teach-back method was effective in addressing anxiety, further refinement might be needed to tackle depression specifically. This might involve integrating supplementary psychological support into the intervention, or perhaps extending the duration of follow-ups to offer patients continuous reassurance and affirmation of their management efforts. The nuanced nature of depression, which can be influenced by a variety of psychosocial factors beyond mere knowledge and understanding, warrants a more comprehensive approach that may include counseling or therapy (29, 30).

Though the teaching-back method exhibited potential benefits in managing postoperative complications and improving the quality of life, these improvements were not statistically significant when compared to the routine nursing group. It was plausible that the short duration between surgery and assessment of quality of life might not have been sufficient for these interventions to exert their full impact. Assessing long-term outcomes and considering other variables such as social support structures, which were known to influence recovery and psychological well-being, might provide further insight into the full ramifications of the teach-back method.

In drawing conclusions from these findings, it was essential to consider certain limitations inherent in the study design. The retrospective nature may introduce bias related to the availability and accuracy of records, though this was mitigated through careful data cleaning and management. Furthermore, the impact of potential confounders, like variations in individual learning styles or external support networks, may not be wholly accounted for. The non-randomized allocation and the potential for the Hawthorne effect are significant limitations that temper the strength of the conclusions. The findings indicate associations that are promising but require confirmation in a prospective, randomized setting. Future studies could benefit from a prospective design and a more diverse patient population to validate and extend these findings.

## 5 Conclusion

In conclusion, the results of this study suggest an association between the teach-back method and enhanced knowledge acquisition, improved self-care management, and reduced anxiety levels among postoperative AD patients. By placing a greater emphasis on patient-centered care and active involvement in their own health education, the teach-back method addresses a critical need in modern healthcare for interventions that not only treat but empower patients. However, the non-randomized design and potential for bias preclude definitive causal inferences. While further research, particularly well-designed randomized controlled trials, is warranted to establish causality and explore the full spectrum of its benefits, particularly in terms of long-term psychological and physical health outcomes, the current findings indicate that this method offers a promising adjunct to routine post-surgical care practices, potentially leading to improved overall patient satisfaction and reduced healthcare costs through better self-management and decreased readmission rates.

## Data Availability

The raw data supporting the conclusions of this article will be made available by the authors, without undue reservation.
